# Targeting purinergic receptors to attenuate inflammation of dry eye

**DOI:** 10.1007/s11302-022-09851-9

**Published:** 2022-02-26

**Authors:** Jia-Ning Wang, Hua Fan, Jian-Tao Song

**Affiliations:** Eye Hospital, China Academy of Chinese Medical Sciences, Beijing, China

**Keywords:** Dry eye disease, Purines, Purinergic receptors, Inflammation

## Abstract

Inflammation is one of the potential factors to cause the damage of ocular surface in dry eye disease (DED). Increasing evidence indicated that purinergic A_1_, A_2A_, A_3_, P2X4, P2X7, P2Y_1_, P2Y_2_, and P2Y_4_ receptors play an important role in the regulation of inflammation in DED: A_1_ adenosine receptor (A_1R_) is a systemic pro-inflammatory factor; A_2AR_ is involved in the activation of the MAPK/NF-kB pathway; A_3R_ combined with inhibition of adenylate cyclase and regulation of the mitogen-activated protein kinase (MAPK) pathway leads to regulation of transcription; P2X4 promotes receptor-associated activation of pro-inflammatory cytokines and inflammatory vesicles; P2X7 promotes inflammasome activation and release of pro-inflammatory cytokines IL-1β and IL-18; P2Y receptors affect the phospholipase C(PLC)/IP3/Ca^2+^ signaling pathway and mucin secretion. These suggested that purinergic receptors would be promising targets to control the inflammation of DED in the future.

## Introduction

Dry eye disease (DED) is one of the most common ocular surface diseases in the world. DED is caused by a variety of etiologies. It is characterized by loss of tear film homeostasis with tear film instability and high osmolarity, ocular surface inflammation and injury, and neurosensory abnormalities [1]. The incidence of DED is increasing every year and has a serious impact on the physical and mental health of the population. The prevalence of DED ranges from 5.5 to 33.7% worldwide, over 30% in China [2]. DED has a great impact on patients’ life and work and even leads to depression. The common clinical treatment method is artificial tears, which cannot solve the root of the problem, and the efficacy is difficult to guarantee. Therefore, it is important to understand the potential therapeutic targets of DED in order to develop effective treatment strategies.

Purinergic receptor was formally proposed as a medical term in 1978. There are four types of P1 receptors (adenosine A_1_, A_2A_, A_2B_, and A_3_), two types of P2 receptors (P2X and P2Y), seven types of P2X receptors (P2X1–7), and eight types of P2Y receptors (P2Y_1_, P2Y_2_, P2Y_4_, P2Y_6_, P2Y_11–14_). Adenosine triphosphate (ATP) binds to these receptors and induces cell-to-cell communication and propagation of inflammation [3]. ATP binds to P2X receptors, which are ligand-dependent ion channels (Na^+^, K^+^, Ca^2+^) to control rapid responses. ATP and adenosine diphosphate (ADP) activate P2Y receptors; this is a second messenger system that acts through G proteins to control the inflammatory release of various neurotransmitters and hormones [4].

**Fig. 1:**
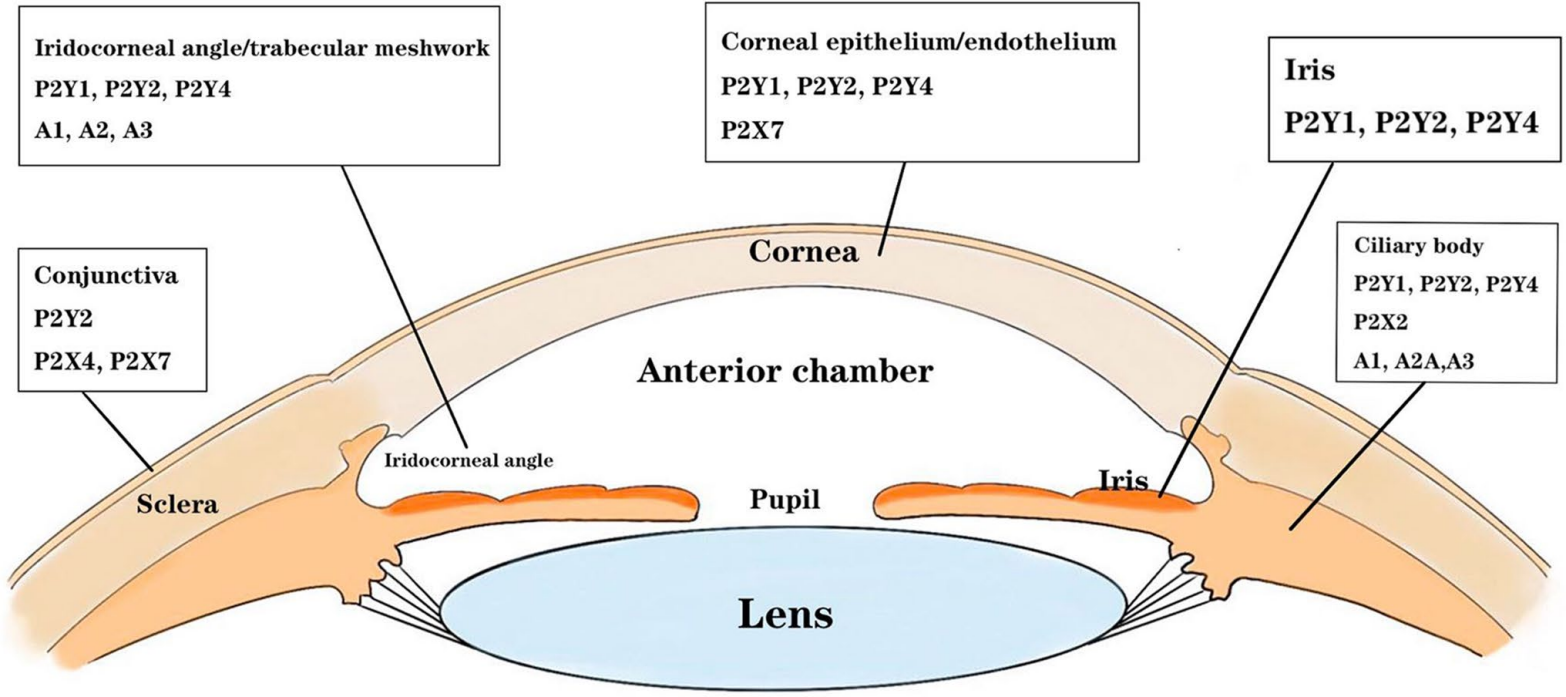
Distribution of purinergic receptors in ocular surface structures

DED is an inflammatory disease that occurs at the ocular surface. The inflammatory components of DED include cells (lymphocytes, macrophages, and T cells etc. [5–7]) and mediators (Th1-related cytokines, matrix metalloproteinases, chemokines, and their receptors etc. [8–11]). Tear film instability, tear hyperpermeability, corneal/conjunctival apoptosis, and inflammation of the ocular surface can create a vicious cycle of inflammation. The relationship between purine receptors and DED is reflected in the widespread presence of purines in ocular tissues such as the cornea, conjunctiva, and lacrimal gland, which play a role in regulating their physiology and pathology [12] (Fig. 1). Purine receptors P2Y_1_, P2Y_2_, P2Y_4_, and P2Y_6_ have been found in corneal epithelial and endothelial cells [13–15]. The expression of purinergic receptors, especially the P2 receptor subtype, is associated with the activation of inflammatory vesicle complexes and the release of proinflammatory cytokines [16]. Adenosine receptors were also found to be highly expressed in inflammatory cells of the cornea [17, 18], and as a result, inflammatory factors act as signaling molecules that bind to their corresponding receptors and cause DED [19]. Undoubtedly, inflammation is one of the most important mechanisms of DED, and purinergic receptors are important pharmacological targets for inflammation therapy. The most widely used treatment for DED is artificial tears. Further treatment emphasizes immunosuppressive or anti-inflammatory drugs that act through the pro-secretory agent pathway with the aim of promoting tear production [20]. These drugs include purinergic P2Y_2_ receptor agonists, mucin secretion stimulants, and so on [21–24]. The therapeutic approach focuses on the underlying pathogenic pathways, which may provide better results. Therefore, in this review, we focus on the molecular mechanism of purine receptors as a potential therapeutic target for DED.

### Adenosine receptors in inflammation

Adenosine is an endogenous nucleoside that is widely distributed in the body and regulates a variety of functions. Among the P1 receptors, A_1R_, A_2AR_, and A_3R_ are mainly involved in the mechanism of inflammation regulation. The biological function of adenosine is mediated through the activation of different G-coupled receptors, A_1R_, A_2AR_, and A_3R_, which inhibit or promote the generation of cyclic adenosine monophosphate (cAMP) in multiple ways. A_1R_ and A_3R_ downregulate CAMP levels and inhibit adenylate cyclase (AC), while A_2AR_ activate AC and promote CAMP production [25]. Activation of A_1R_ promoted the expression of pro-inflammatory factors, while A_2AR_ and A_3R_ inhibited the expression of pro-inflammatory factors.

#### *A*_*1*_* receptor*

Ponnoth Dovenia S et al. observed the effects of nebulized adenosine inhalation on vascular reactivity and inflammatory responses in A_1R_ knockout (A_1_KO) and corresponding wild-type (A_1_WT) mice and demonstrated that A_1R_ is a systemic pro-inflammatory factor [26]. Furthermore, the impact of adenosine on inflammatory regulation suggests that the adenosine receptors (ARs) can be activated or inactivated by the use of selective agonists or antagonists, which includes a potential contribution to inflammation-like diseases. Based on this theory, a newly synthesized in-house compound, 8-chloro-9-ethyl-2-phenethoxyadenine, was proposed as a candidate for inflammation therapy [27]. Studies of ophthalmologically relevant drug for A_1R_ have focused on retinal diseases [28], and because the drug did not pass phase III clinical trials [29], it was regretfully terminated. The A_1R_ antagonist has little prospect as a DED candidate because it increases Intraocular pressure in mice and rabbits [25].

#### A_2A_ receptor

A_2AR_ exerts its anti-inflammatory effects primarily by inhibiting the secretion of pro-inflammatory cytokines after the activation of inflammatory responses. Ko Il-Gyu proposes that A_2AR_ may be involved in the activation of the MAPK/nuclear factor-kappa B (NF-kB) pathway as an important neuromodulator. NF-kB is stimulated by various factors such as cytokines and cellular stress, and activation (phosphorylation) of mitogen-activated protein kinase (MAPK)/NF-kB may promote the expression of multiple pro-inflammatory cytokines, resulting in inflammation, which is one of the mechanisms [30, 31]. In a mouse model of traumatic optic neuropathy (TON), Ahmad et al. reported that A_2AR_ agonist treatment significantly reduced the expression of inflammatory mediators (such as intercellular adhesion molecule-1, IL-6, and TNF-α) and microglial marker IBA-1 [32].The protective role of A_2AR_ activation in diabetic retinal inflammation has been very well documented, and studies suggest that A_2AR_ agonists produce anti-inflammatory effects by blocking ERK activation and subsequent cytokine release [32, 33]. It is also a good inspiration for drug research in DED. Istradefylline, a specific A_2AR_ antagonist, decreased choroidal neovascularization [34]. US FDA approval of the istradefylline for treatment of Parkinson’s disorders. This provides the safety profile of the A_2AR_ antagonist in human, and it facilitates to develop ophthalmic drugs about A2 for the future.

#### A_3_ receptor

A_3R_ is involved in a number of different intracellular signaling pathways, and the A_3R_ was reported by Avni Isaac et al. to be highly upregulated in inflammatory cells [35]. Recently, the role of A_3R_ as a regulator of inflammation has become clear, making it an attractive target for the treatment of inflammatory diseases. A_3R_ is linked to the inhibition of adenylate cyclase and the regulation of MAPK pathways, leading to the regulation of transcription [36]. CF101 (N6-(3-iodobenzyl)-5′-Nmet-hylcarboxamidoadenosine), an A_3R_ agonist for the treatment of inflammatory diseases, downregulates the NF-kB signaling pathway, thereby inhibiting pro-inflammatory cytokines (TNF-α, IL-6, IL-12) and macrophage inflammatory proteins (MIPs-1a, MIP-2), resulting in apoptosis of inflammatory cells [37, 38]. CF101 exerted powerful anti-inflammatory effects in animal studies [39]. A randomized, multicenter, double-masked, placebo-controlled, parallel-group Phase II clinical study investigated the safety and efficacy of CF101 in the patients with moderate-to-severe DED. Patients aged ≥ 18 years diagnosed with moderate to severe DED were selected, and they were randomly assigned to receive either a 1 mg CF101 pill (oral) or matching vehicle-filled placebo pills twice daily for 12 weeks. Patients also received individually packaged preservative-free artificial tears as adjunctive therapy for up to eight times daily for the duration of the trial. Patients returned for clinical evaluation and new study drug supply at weeks 2, 4, 8, and 12, with a final follow-up evaluation at week 14 after 2 weeks of discontinuation. The results showed that 84.6% of patients had more than 25% improvement in corneal staining, and there were statistically significant differences between the CF101 and placebo treatment groups. The mean change in BUT from baseline was improved by CF101 over the placebo group. No serious adverse events were identified throughout the study. The most common adverse events included constipation, headache, palpitations, pruritus, abdominal pain, myalgia, fatigue, and dry mouth. The results of this study showed that oral administration of CF101 1 mg twice daily was well tolerated and demonstrated an excellent safety profile and sustained anti-inflammatory effect for up to 18 months. The anti-inflammatory effects of CF101 may influence the pathogenesis of distant disease and may serve as a novel approach to treating the cause of the disease, rather than just the symptoms [35]. Also, CF101 has demonstrated efficacy and safety in other inflammatory diseases such as psoriasis arthritis in clinical trials, and it is expected that oral small molecule drug CF101 will provide new prospects for the treatment of DED. However, DED is a chronic disease that lasts for a long time. Therefore, longer studies using CF101 in patients with DED are still needed to know long-term efficacy and safety of this drug.

### P2X receptors in inflammation

#### P2X4 receptor

Inflammation and inflammasomes caused by P2X4R activation have also been implicated in the pathogenesis of DED. Components of NLRP3 are found in conjunctival capsular cells, which are punctate receptors that sense pathogens and other triggers, and are highly expressed in moist mucosa [40]. NLRP3 is a member of a multiprotein complex known as the NLRP3 inflammasome, which may activate IL-1b secretion and promote inflammation of the conjunctiva through the caspase-1 pathway. A study by Li Lin et al. showed that activation of P2X4R by ATP activates pro-inflammatory cytokines [41]. P2X4R may play a role in the initiation and activation of the NLRP3 inflammasome [40]. The above studies indicate that P2X4R activation may contribute to the development and worsening of DED. Therefore, it is important to inhibit its activation under inflammatory conditions. Minimizing the overexpression of P2X4R may be a target for the treatment of DED.

#### P2X7 receptor

P2X7R is an important target in the pathogenesis and treatment of DED. The ATP-gated P2X7R is a twice-transmembrane ion channel that is permeable to Na^+^, K^+^, and Ca^2+^. Activation of P2X7R activates the inflammasome and promotes the release of the proinflammatory cytokines IL-1β and IL-18 [42, 43]. When P2X7R is activated, cells are permeabilized with monovalent and divalent cations (Na^+^, K^+^, Ca^2+^) and permeability. Rapid opening of potassium-selective channels results in a rapid decrease in intracellular potassium levels and the release of proinflammatory cytokines [44]. The inward flow of Ca^2+^ ions compensates for the outward flow of K^+^ ions, resulting in P2X7R-induced intracellular K^+^ ion loss or increased cytoplasmic Ca^2+^ ions thereby activating caspase-1. This activated caspase-1 further leads to rapid activation and release of the pro-inflammatory cytokine IL-1β. The subsequent increase in the concentration of IL-1β induces inflammation, which in turn induces other inflammatory mediators such as procysteine aspartate-1, nitric oxide synthase, cyclooxygenase-2, TNF-α, phospholipase D, phospholipase A2, NF-kB, and MAPK [45]. Furthermore, P2X7R plays an important role in the regulation of wound healing by regulating corneal integrity and corneal epithelial cell migration [46]. DED is a disease of ocular surface inflammation and damage (including corneal epithelial damage) by multi-factor, inflammation aggravates ocular surface damage, and damage aggravates ocular surface inflammation, forming a vicious circle. Based on the role of P2X7R in inflammation and corneal epithelial damage, it is worth to focus on the development of antagonists and agonists of P2X7R.

### P2Y receptors in inflammation and mucin

**Fig. 2:**
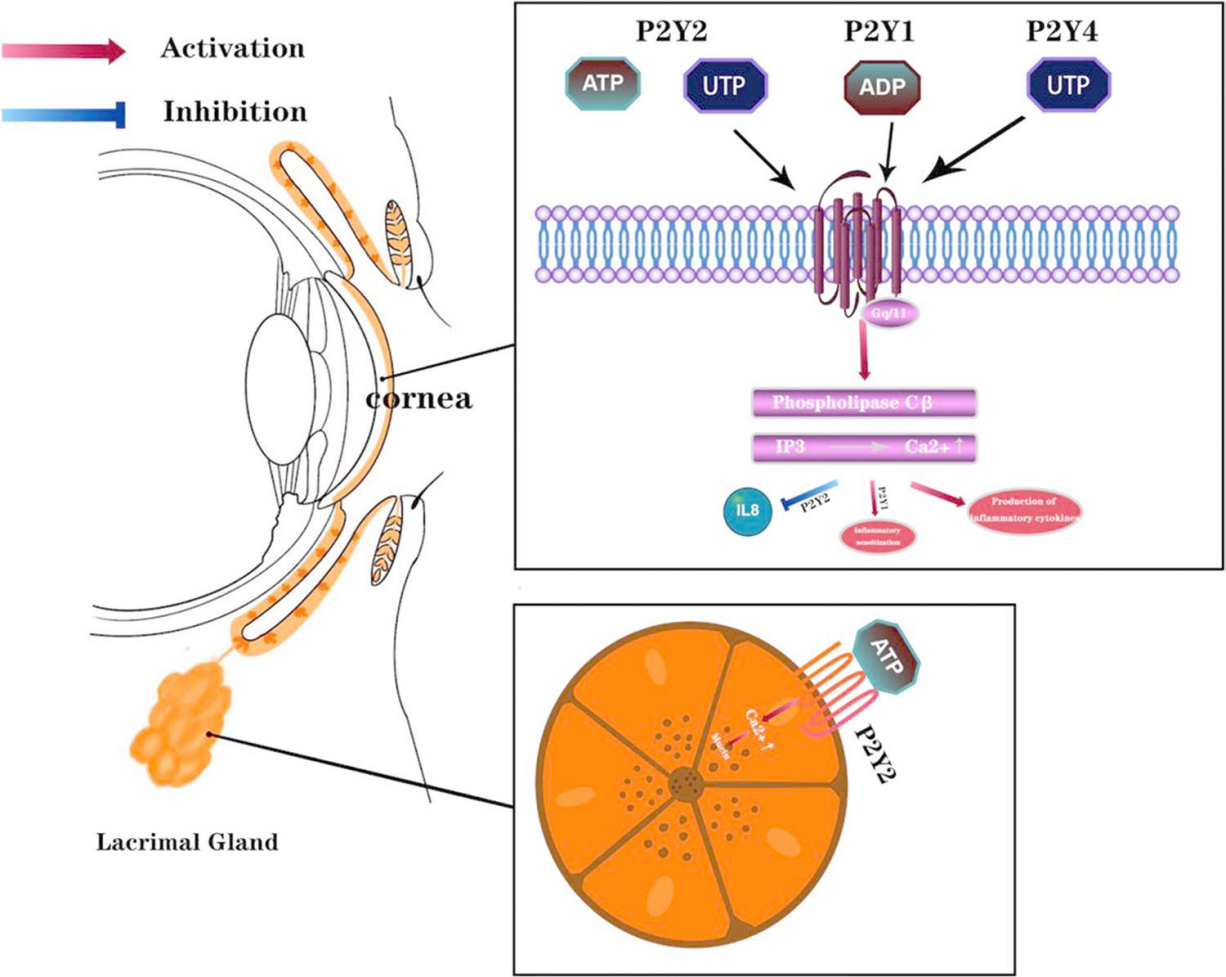
P2Y_1R_, P2Y_2R_, and P2Y_4R_ are coupled to GQ and activate the phospholipase C (PLC)/IP3/Ca2 + signaling pathway, P2Y_1_ is involved in inflammatory sensitization, P2Y2 receptor agonist induced IL-8 release, P2Y4R is involved in inflammatory cytokine production (top), P2Y_2_ receptor stimulated Ca^2+^ concentration increase in lacrimal gland follicles leading to mucin secretion (bottom)

P2Y_1_, P2Y_2_, and P2Y_4_ receptors are found in the corneal epithelium and endothelium [13, 14]. They can mediate Ca^2+^ mobilization after being activated by ATP or uridine 5′-triphosphate (UTP), which results in downstream signaling to promote inflammation. According to the G protein coupling properties of P2YRs, P2Y_1R_, P2Y_2R_, and P2Y_4R_ can be coupled to GQ to activate the phospholipase C (PLC)/IP3/Ca^2+^ signaling pathway. P2Y_1R_ is involved in inflammatory sensitization [47]. High extracellular NaCl induces priming of the NLRP3 inflammasome in RPE cells, in part via P2Y_1_R signaling [48]. P2Y_2R_ agonist induced IL-8 release and immune-mediated inflammation of the mucosa characterized by the release of interleukin IL-8[49]. P2Y_4R_ signaling is involved in the production of pro-inflammatory cytokines via PI3K/Akt and ERK1/2-dependent pathways, and inhibition of P2Y_4R_ expression significantly suppresses the production of pro-inflammatory cytokines [50]. Furthermore, G protein-coupled receptor kinase (GRK) 2 can induce and regulate the intensity and duration of inflammation [51]. Thus, the P2Y_1_, P2Y_2_, and P2Y_4_ receptors may be involved in the pathogenesis of inflammation in DED. On the other hand, P2Y_2R_ promotes mucus secretion from conjunctival gland vesicle cells by activating intracellular signals and specific transcription factors, and upregulates the expression of mucus genes [52]. Mucus proteins also reduce damage to mucosal epithelial cells in dry environments by lubricating the ocular surface and reducing friction. Mucin also plays an important role in DED due to its immune functions, such as strengthening the epithelial barrier, mediating various antimicrobial components, preventing pathogens from adhering to the ocular surface, and assisting in the removal of pathogenic microorganisms and cellular debris from the mucosal surface by aggregation [53, 54]. Studies have shown that purine P2Y_2R_ promotes an increase in Ca^2+^ concentration in cell membranes, which results in the secretion of mucin [55, 56] (Fig. 2). Diquafosol tetrasodium acts as a P2Y_2R_ agonist, activating P2Y_2R_ on the ocular surface to improve the quantity and quality of tear fluid in patients with DED through a novel mechanism that promotes aqueous humor transport, conjunctival epithelial mucus secretion, lipid production, and cell survival [57]. Nucleotides such as ATP and UTP are known to regulate processes such as tear secretion, wound healing, and prevention of surface infection. Diquafosol is a dinucleotide derivative of UTP [58]. Tadahiro Murakami et al. analyzed the ion transport system of the conjunctiva, another mechanism of tear secretion, using the Ussing chamber method. Their findings suggest that Diquafosol directly stimulates functional P2Y_2R_ in conjunctival epithelial cells, increasing fluid flow and allowing Cl^−^ secretion from the cell membrane to the mucosal side [59]. Furthermore, Diquafosol inhibits ERK-p90RSK-mediated apoptosis and enhances cell survival by inhibiting the NF-kB signaling pathway. It was also shown that Diquafosol has anti-ROS activity and that both ROS production and expression of the inflammatory mediator IL-1b and TNF-α were suppressed by Dictyostelium japonicum [57]. Overall, the role of P2YR in the molecular pathway of DED cannot be ignored. Systematic summary information is shown in Table 1.

**Table 1:** The mechanism of inflammation and ocular disease mediated by purinergic receptors

Receptor subtype	Molecular mechanism	Method/experimental models	Type of disease	Agonist/antagonist
A_1R_	Attenuates damage caused by NMDA, inhibits the glutamate-induced calcium influx, and increases the AH outflow through secretion of MMP-2 [25]	The HTM-3 cell line primary cultures of BTM cells [25]	Primary open-angle glaucoma [28, 29]	8-chloro-9-ethyl-2-phenethoxyadenine [27]
A_2AR_	Inhibits the MAPK pathway [30] and inhibits NF-kB and the JAK/STAT pathways [32]	TON mouse model [32]	TON [32]	Dimethyl sulfoxide or CGS21680 [32]
A_3R_	Inhibits the MAPK pathway[36], inhibits the NF-kB pathway[37], and inhibits TNF-α, IL-6, IL-12, MIPs-1a, MIP-2 [38]	A randomized, multicenter, double-masked, placebo-controlled parallel-group Phase 2 clinical study [35]	DED [35]	CF101 [35]
P2X4R	Activates NLRP3 inflammatory vesicle [40]	Goblet cells from rat and human conjunctiva [40]	The innate immune response in the conjunctiva [40]	-
P2X7R	Induces intracellular K^+^ ion loss or increases cytoplasmic Ca^2+^ ions, thereby activates caspase-1 release IL-1βand other inflammatory mediators [44, 45], forms organized stroma, and regulates a number of proteins [46]	Bac1 murine macrophages [45] P2X7 knockout and WT mice [46]	Conjunctivitis [45] DED [46]	Extracellula-r ATP or nigericin [45]
P2Y_1R_	Couples to GQ to activate the PLC/IP3/Ca^2+^ signaling pathway[47] and induces priming of the NLRP3 inflammasome in RPE cells [48]	Adult male wild type C57/Bl6 mice [47] RPE cells [48]	Inflammation [47] AMD [48]	-
P2Y_2R_	Couples to GQ to activate the PLC/IP3/Ca^2+^ signaling pathway and induces IL-8 release [49], increases Ca^2+^ concentration in cell membranes, resulting in the secretion of mucin [55], inhibits ERK-p90RSK-mediated apoptosis, enhances cell survival, and inhibits the NF-kB signaling pathway [57]	HEECs cultured with the use of an ALI system [49] NHMEE cells [55] Human corneal epithelial cells [57]	Immune-mediated mucosal inflammation [49] DED [55, 57]	P2Y_2_ receptor agonist [49, 55] Diquafosol [57]
P2Y_4R_	Produces pro-inflammatory cytokines via PI3K/Akt and ERK1/2 pathways [50]	C57BL/6 mice P2Y_4R_ knockdown mice/astrocytes/murine hippocampal neuron HT-22 cells/human glioma U261 cells [50]	Human immunodeficie-ncy diseases [50]	-

*A*_*1R*_ A_1_ receptor, *NMDA* N-methyl-D- aspartate, *ARs* adenosine receptors, *TM* trabecular meshwork, *AH* aqueous humor, *MMP-2* matrix metalloproteins, *HTM-3* human trabecular meshwork, *BTM* bovine trabecular meshwork, *A*_*2AR*_ A_2A_ receptor, *MAPK* mitogen-activated protein kinase, *NF-kB* nuclear factor-kappa B, *JAK/STAT* janus kinase/signal transducer and activator of transcription, *TON* traumatic optic neuropathy, *A*_*3R*_ A_3_ receptor, *MIP* macrophage inflammatory proteins, *DED* dry eye disease, *P2X4R* P2X4 receptor, *NLRP3* pyrin domain-containing protein 3, *P2X7R* P2X7 receptor, *WT* wild-type, *ATP* Adenosine triphosphate, *P2Y*_*1R*_ P2Y_1_ receptor, *NLRP3* pyrin domain-containing protein 3, *RPE* retinal pigment epithelial, *AMD* age-related macular degeneration, *P2Y*_*2R*_ P2Y_2_ receptor, *PLC* phospholipase C, *IL* interleukin, *HEECs* human esophageal epithelial cells, *ALI* air–liquid interface, *NHMEE* normal human middle ear epithelial, *P2Y*_*4R*_ P2Y_4_ receptor

## Conclusion and prospects

This review summarizes the possible molecular mechanisms by different purinoceptors that mediate DED. A_1_, A_2A_, A_3_, P2X4, P2X7, P2Y_1_, P2Y_2_, and P2Y_4_ have been studied in ocular disease and inflammation, and the results of these studies are more valuable for mechanisms and therapy of DED. Other purines are not discussed in this review since their role in ocular disease and inflammation is rarely reported. Purinergic receptors act on inflammation from different targets and are effective in reducing inflammation and ocular surface damage in DED. Currently, the treatment for DED is mainly artificial tears and anti-inflammatory drugs. However, such anti-inflammatory drugs can cause ocular surface damage. Purinergic receptors play an active role in inflammation and ocular surface damage in DED. Therefore, focusing on purine receptors, the development of related drugs for the treatment of DED has broad prospects.

A new approach called polypharmacology has been gaining attention in the last decade. Polypharmacology is the use of a single drug to modulate multiple targets associated with a single disease pathway. In this regard, drugs that reach multiple sensitive targets (multi-targeted drugs) may have higher efficacy and can reduce the disadvantages usually associated with the use of combination therapies [60, 61]. Bevan Nicola et al. designed an anti-inflammatory agent, GW328267X, an A_2AR_ agonist, and A_3R_ antagonist [62]. Hou Xiyan et al. designed a new series of dual-acting hA_2AR_ agonists and hA_3R_ antagonists for the treatment of inflammatory diseases [63]. Therefore, we believe that in the near future, multi-targeted drugs will be developed in the field of DED, and we can expect better effects, even the development of lubricant and purine combination drug dual component eye drops. In addition, since there are so many ATP-releasing tissues and excess purine receptors in the eye, they may behave off-target, especially when released in excess in pathological or inflammatory conditions, probably due to the interaction of purine signaling systems in different ocular regions, leading to problems in ocular diseases; this is thought to be the case. This review not only contributes to a better understanding of purinergic receptors, but also provides a basis and direction for better treatment of DED in the future by altering the molecular mechanisms associated with purinergic receptors.

## Data Availability

Not applicable.
